# Health Tract, No. 188

**Published:** 1864-02

**Authors:** 


HEALTH TRACT, NO. 188.

### CONVENIENT KNOWLEDGE.

MUTTON can be produced more cheaply than any other meat, and yet it is quite as nutritious as beef, while it has not so much waste. Pugilists as often "train" on mutton as on beef.

A CELLAR which opens inside a dwelling should be kept as faultlessly clean all the year round as any other part of the house, because its atmosphere is constantly ascending, and impregnates every room in the house with its own odors. In reality, there ought not to be any cellar under any dwelling.

SQUEAKING boots or shoes are a great annoyance, especially in entering a sick-room, or a church after the services have commenced; the remedy is, to boil linseed oil and saturate the soles with the same.

NEURALGIA of the severest character is sometimes removed by painting the parts two or three times a day with a mixture composed of half an ounce of the Tincture of Iodine, and half a drachm of the Sulphate of Morphine.

LINIMENT. One of the most powerful liniments for the relief of severe pain, is made of equal quantities of spirits of hartshorn, sweet oil, and chloroform; dip into this a piece of cotton cloth doubled, about the size of a silver dollar, lay it on the spot, hold a handkerchief over the spot, so as to confine the fumes, and the pain immediately disappears. Do not let it remain on over a minute. Shake it well just before using, and keep the bottle very closely stopped.

CHEMICAL AGENCIES. If a single drop of sweet oil comes in contact with half an ounce of the chloride of nitrogen, it would explode with such power as to shiver a house to atoms.

THE highest wave does not exceed twenty feet, and a man may easily "ride them"—and thus prevent himself from drowning—by throwing himself on his back, just keeping his nose above water, and joining his hands under the water.

DENTISTRY. It is becoming fashionable to have teeth extracted while the person is in a state of insensibility, caused by inhaling nitrous oxide gas, commonly known as "laughing gas." When first discovered it was used freely, but in some cases dangerous results followed, as testified to by Sir Humphry Davy, Professor Silliman, Pereira, Berzelius, Ayston, and others. But from the fact that no such ill results have been observed for many years past, although it has been taken by scores of thousands, it may be reasonably concluded that a purer quality is now prepared, and that its administration is safe.

FOOD. The most easily digested articles of food as yet known, are sweet apples baked, cold raw cabbage sliced in vinegar, and boiled rice; the most indigestible are suet, boiled cabbage, and pork; the former requires an hour, the latter five.

